# Naringin from Coffee Inhibits Foodborne *Aspergillus fumigatus* via the NDK Pathway: Evidence from an In Silico Study

**DOI:** 10.3390/molecules28135189

**Published:** 2023-07-04

**Authors:** Shashanka K. Prasad, Smitha S. Bhat, Olga Koskowska, Jiraporn Sangta, Sheikh F. Ahmad, Ahmed Nadeem, Sarana Rose Sommano

**Affiliations:** 1 Department of Biotechnology and Bioinformatics, JSS Academy of Higher Education and Research, Mysuru 570 015, India; smithasbhat@jssuni.edu.in; 2 Plant Bioactive Compound Laboratory, Faculty of Agriculture, Chiang Mai University, Chiang Mai 50100, Thailand; jiraporn_sangta@cmu.ac.th; 3 Department of Vegetable and Medicinal Plants, Institute of Horticulture Sciences, Warsaw University of Life Sciences—SGGW, 16602-787 Warsaw, Poland; olga_kosakowska@sggw.edu.pl; 4 Interdisciplinary Program in Biotechnology, Graduate School, Chiang Mai University, Chiang Mai 50200, Thailand; 5 Department of Pharmacology and Toxicology, College of Pharmacy, King Saud University, Riyadh 11451, Saudi Arabia; fashaikh@ksu.edu.sa (S.F.A.); anadeem@ksu.edu.sa (A.N.); 6 Department of Plant and Soil Sciences, Faculty of Agriculture, Chiang Mai University, Chiang Mai 50200, Thailand

**Keywords:** coffee, *Aspergillus fumigatus*, nucleoside diphosphate kinase, in silico, antifungal

## Abstract

In the tropics, coffee has been one of the most extensively cultivated economic crops, especially Arabica coffee (*Coffea arabica* L.). The coffee pulp, which includes phytochemicals with a proven antifungal action, is one of the most insufficiently utilized and neglected byproducts of coffee refining. In the current experiment, we carried out in silico screening of the isolated Arabica coffee phytochemicals for antifungal activity against *Aspergillus fumigatus*: a foodborne fungus of great public health importance. As determined by the molecular docking interactions of the library compounds indicated, the best interactions were found to occur between the nucleoside-diphosphate kinase protein 6XP7 and the test molecules Naringin (−6.771 kcal/mol), followed by Epigallocatechin gallate (−5.687 kcal/mol). Therefore, Naringin was opted for further validation with molecular dynamic simulations. The ligand–protein complex RMSD indicated a fairly stable Naringin-NDK ligand–protein complex throughout the simulation period (2–16 Å). In ADME and gastrointestinal absorbability testing, Naringin was observed to be orally bioavailable, with very low intestinal absorption and a bioavailability score of 0.17. This was further supported by the boiled egg analysis data, which clearly indicated that the GI absorption of the Naringin molecule was obscure. We found that naringin could be harmful only when swallowed at a median lethal dose between 2000 and 5000 mg/kg. In accordance with these findings, the toxicity prediction reports suggested that Naringin, found especially in citrus fruits and tomatoes, is safe for human consumption after further investigation. Overall, Naringin may be an ideal candidate for developing anti-*A. fumigatus* treatments and food packaging materials. Thus, this study addresses the simultaneous problems of discarded coffee waste management and antifungal resistance to available medications.

## 1. Introduction

*Aspergillus fumigatus (A. fumigatus)*, a saprophytic and thermophilic ascomycete fungus, reportedly causes the most serious diseases in humans among all the foodborne Filamentous Fungal Human Pathogens (FFHPs) [1]. Accounting for as much as 85–95% of the invasive aspergillosis instances, *A. fumigatus* is regarded as the most dangerous fungal pathogen to be found in food [2]. Within the *Aspergillus* species, *A. fumigatus* is regarded as the biggest threat when trailed by *A. flavus*, *A. niger*, *A. terrus* and *A. nidulans*. Invasive aspergillosis caused by the *A. fumigatus* is one of the foremost reasons for decease in those patients suffering from hematological malignancies and those undergoing chemotherapy [3] and hematopoietic cell transplantations [4]. This deadly human pathogen can be found in baked goods, beans, beverages and chocolates–staple cereals such as maize, rice, barley, and wheat–dairy, fruits, herbs and spices, meat, fish, and eggs, nuts, seeds, and vegetables, with the second highest prevalence only next to *A. flavus* [1]. Gliotoxin (GTX), a dipeptide virulence factor that is mainly produced by *A. fumigatus*, is known for the creation of reactive oxygen species (ROS) through reactions associated with redox cycling, which led to immunosuppression and necrosis [5,6]. In addition, GTX has been reported to show an alteration in tight junction structures and to cause neuronal damage by impairing the human blood–brain barrier via cytotoxicity towards the astrocytes [7]. GTX, at concentrations lower than 250 ng/mL, was found to inhibit inflammatory cell activation and signal transduction between the leukocytes and other phagocytic white blood cells at lower concentrations [8]. Meanwhile, at higher concentrations, it reportedly induced leukocyte apoptosis [9]. Owing to these immune-suppressing capabilities of the toxin, Ráduly Z et al. (2020) indicated that *A. fumigatus* had the potential to evade the immune responses and enhance AIDS and substance-induced immunodeficiencies due to GTX poisoning [10]. Fumagillin, first isolated in 1949 from *A. fumigatus*, is a mycotoxin encoded in chromosome 8 of the fungus [11,12,13]. This toxin was found to bind to the methionine aminopeptidase (MetAP) type 2 enzyme, inactivating it irreversibly [14], thereby inhibiting neutrophil functions [15], eryptosis [16], damaging lung epithelia [17], and anti-angiogenesis [14]. Given the potential hazard of *A. fumigatus* contamination in food, there is a mounting necessity for food-grade antifungal drugs and preservatives to address both public health and food security concerns.

With the increasing instance of *A. fumigatus* infections and reports of antifungal resistance, there has been a necessity to identify and develop novel antifungal agents. With the focus now more oriented toward understanding the molecular intricacies underlying the propagation of the fungus, there are new avenues open to identify potential drug targets which could inhibit fungal growth and development. The *A. fumigatus* nucleoside-diphosphate kinase (NDK) enzyme catalyzes the final step of nucleotide biosynthesis by phosphorylating the nucleoside-diphosphates (NDPs) to nucleoside-triphosphates (NTPs) is an essential enzyme for the survival of the fungus. Regarding the antifungal activity evaluation, the biosynthesis of nucleotide purine and salvage pathways have been extensively studied against fungi of clinical importance, such as *C. neoformans*, *C. albicans*, and *A. fumigatus*. They all indicated a correlation between the disruption of the purine biosynthesis pathway and the subsequent attenuation of virulence in the fungi [18,19]. NDK was found to exhibit the preferential nucleoside selectivity of adenine nucleosides over their cytosine counterparts, making them an attractive target for novel antifungal agents [20] and making them the target of choice for this study.

Coffee, a tropical cash crop found across the continents of Asia, America, and Africa, is a beverage of choice globally. Coffee has been generated in quantities as high as 171 million (60 kg) bags in the current year, and 98 million bags of *Coffea arabica* alone were cultivated in the year 2022 [21]. Coffee phytochemicals have been reported for their bioactivity ranging from antioxidant to neuroprotective activities [22]. Calheiros D et al. (2023) reported the antifungal activity of coffee against dermatophytes belonging to *Candida* spp. and *Trichophyton* spp. [23]. Another study focused on the use of fresh and spent Robusta coffee on the *Areca catechu* leaf sheath for the packaging of different foods, concluding that coffee phytochemicals exhibited significant antifungal activity against *Aspergillus* spp., *Penicillium* spp., and *Eurotium* spp. [24]. Sangta J et al. (2021) conducted an evaluation of Arabica coffee pulp phytochemicals for their antifungal activity against *Alternaria brassicicola*, *Pestalotiopsis* sp. and *Paramyrothecium breviseta*, which were found to infest vegetables, fruits, and coffee, respectively. The polyphenol composition of the above study comprised flavonoids such as epigallocatechin gallate (31.8%), naringin (9.63%), epicatechin gallate (8.66%), catechin (2.2%), gallocatechin gallate (0.12%), quercetin (5.42%), and phenolic acids and caffeine alkaloids such as caffeic acid (68.1%), caffeine (21.59%), p-coumaric acid (11.04%), rosmarinic acid (6.41%), o-coumaric acid (6.24%), and gallic acid (2.41%) [25]. All the above-identified phytochemicals were used to prepare the ligand library for the current study.

Over the past few decades, computational techniques have developed into useful tools for drug-related investigations. Bioinformatics is the application of these techniques to biochemical phenomena. Using bioinformatics, drug design can be more precisely and accurately guided by the knowledge of new chemical structures, functions, and targets. Since 1980, the concept of the “rational use of drugs” (structure-guided drug design) has gained popularity. It involves the creation of target proteins and small molecules in silico as the basis for novel medication development [26]. The advantages of computational approaches include minimal costs and quick information gathering. To streamline the process of scientific inquiry, they can be supplemented by an experimental study. In this manner, bioinformatics aids in broadening the scope of experimental findings and vice versa. The entire compound library can be evaluated against the drug target in high throughput screening. Secondary assays are necessary to confirm the location of action for drugs in more complicated systems, such as a complex biological cell-based test, whose activity depends on the target [27].

High throughput and other compound screens are designed and carried out in order to uncover compounds that interact with the therapeutic target. Chemistry programs are then executed to strengthen the potency, selectivity, and physiochemical nature of the molecule. Additionally, information is still being gathered to back up the claim that treating illnesses by intervening at the drug target could be successful. A number of naturally available sources (plants and microbes) have been thus investigated via computational techniques for their various bioactive nature, including antibacterial, antiviral, antifungal and anti-cancer properties [28,29,30,31,32,33]. Many such identified phytocompounds, with further validation, could potentially be developed into novel drugs.

## 2. Results

### 2.1. Molecular Docking

**Figure 1:**
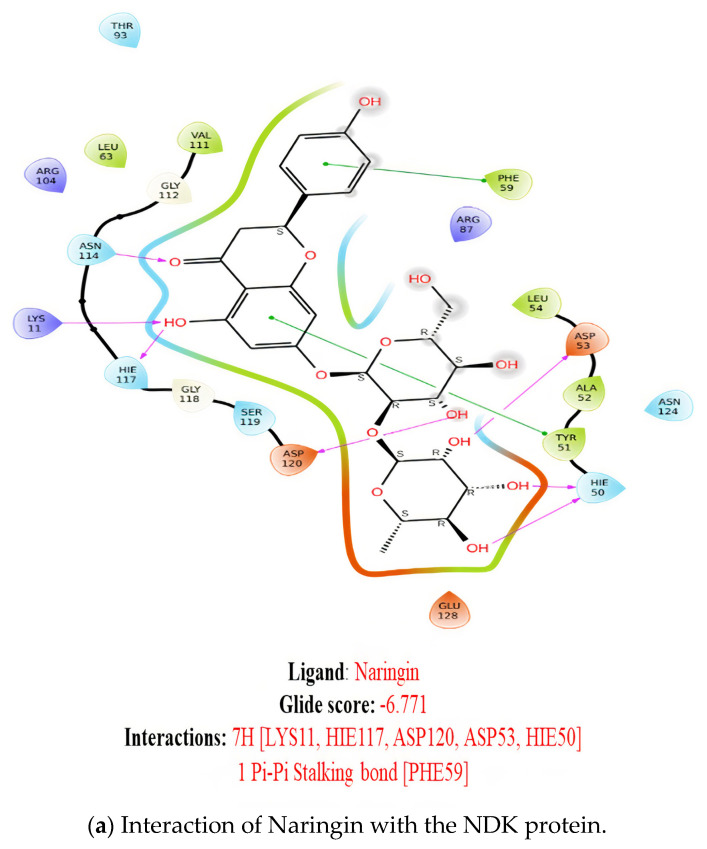

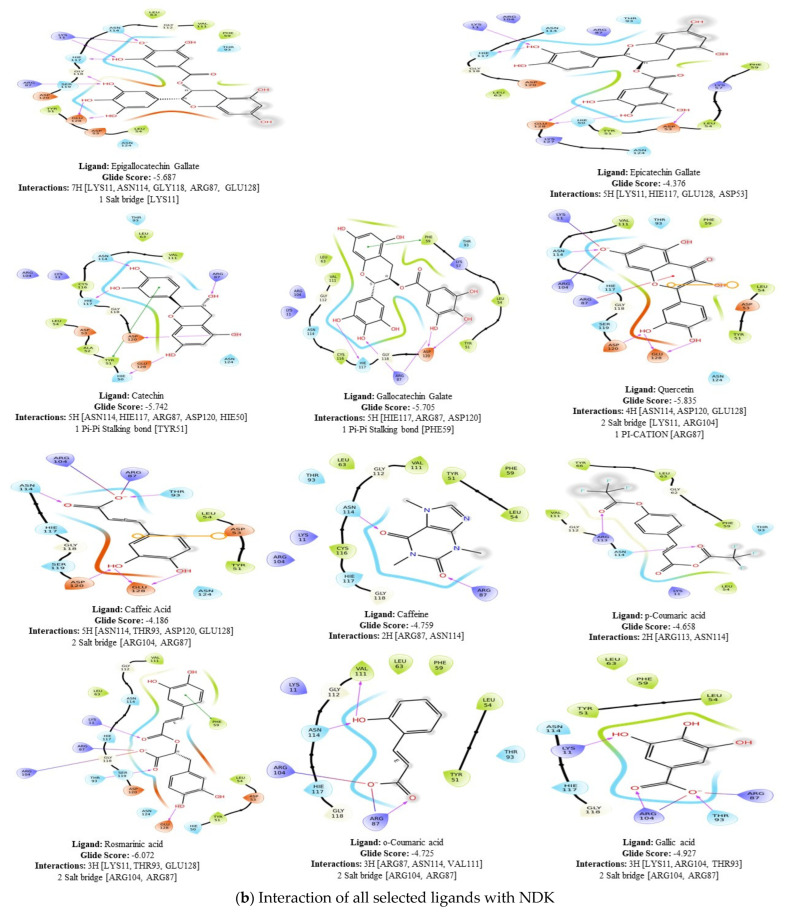
The 2D molecular docking interactions of the selected coffee phytochemicals with the nucleoside diphosphate kinase (NDK) protein 6XP7.

The molecular docking interactions of the library compounds (Figure 1) indicated the best interactions occurring between the NDK protein 6XP7 and the test molecules naringin with a formation of 7 hydrogen bonds for the amino acids LYS11, HIE117, ASP120, ASP53, HIE50 amino acids, and 1 Pi-Pi Stalking bond with the PHE59. A glide score of −6.771 kcal/mol was recorded for this interaction, followed by epigallocatechin gallate with a lower glide score of −5.687 kcal/mol, 7 hydrogen bonds [LYS11, ASN114, GLY118, ARG87, GLU128] and 1 salt bridge [LYS11] with the protein. It is interesting to note that although both ligands displayed the same number of interactions with the chosen NDK protein [7], their glide g scores were varied. The other molecules, including epicatechin gallate, caffeine, catechin, gallocatechin gallate, quercetin, caffeic acid, p-coumaric acid, rosmarinic acid, o-coumaric acid, and gallic acid, were each found to have interactions involving 5H and −4.376 kcal/mol, 5H and −5.742 kcal/mol, 5H and −5.705 kcal/mol, 4H, 1 pi-cation and −5.835 kcal/mol, 5H, 2 salt bridge and −4.186 kcal/mol, 2H and −4.759 kcal/mol, 2H and −4.658 kcal/mol, 3H, 2 salt bridge and −6.072 kcal/mol, 3H, 2 salt bridge and −4.725 kcal/mol, and 3H, 2 salt bridge and −4.927 kcal/mol for the interactions and glide scores, respectively.

### 2.2. Molecular Dynamics Simulation

Of all the interactions observed above, the best-docked complex of NDK against Naringin opted for an MD simulation. This simulation was carried out for a total duration of 100 ns, throughout which the stability of the protein, ligand (Naringin), and the protein–ligand complex was observed via parameters such as the Root Mean Square Deviation and fluctuation, amino acid contacts between the protein and ligand, changes occurring in the structure of the protein, and the properties of Naringin.

### 2.3. RMSD

**Figure 2:**
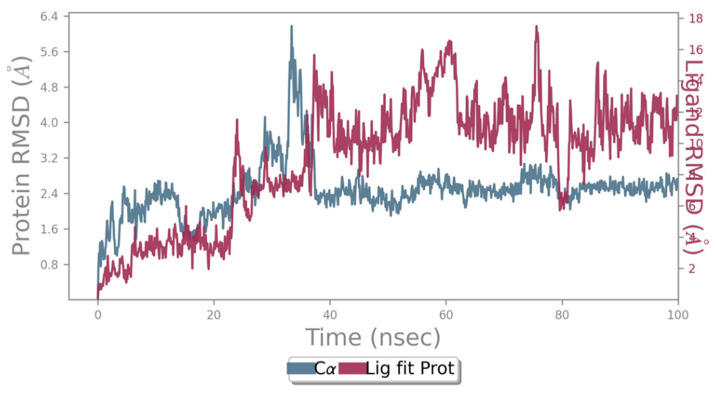
Ligand-Protein RMSD of the Naringin–NDK complex.

The Root Mean Square Deviation (RMSD) is a quantitative measure that can understand the similarity between two superimposed atomic coordinates. In this study, the protein RMSD value ranged between 0.8 and 3.2 Å, with one outlying peak at 6.4 Å indicating a stable protein throughout the 100 ns simulation period (Figure 2). The ligand–protein complex RMSD ranged between 2 and 16 Å, indicating a fairly stable Naringin-NDK ligand–protein complex throughout the 100 ns simulation period (Figure 2).

### 2.4. Protein RMSF

**Figure 3:**
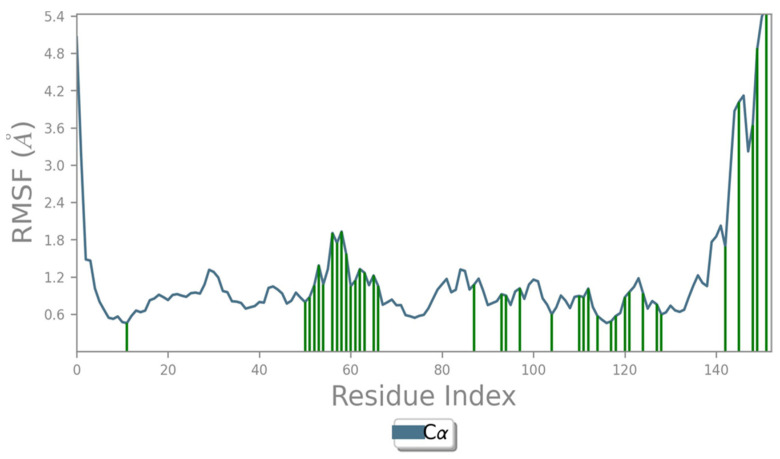
RMSF of the NDK Protein.

The Root Mean Square Fluctuation (RMSF) correlated to the time-average position of a particle, indicating the average deviation undergone by the particle over a particular period from its reference position. The point of interaction between amino acids was mostly stabilized between 0.6 and 1.8 Å, with fluctuating high peaks at 4.2, 4.8 and 5.4 Å (Figure 3).

### 2.5. Total Secondary Structures (SSE)

**Figure 4:**
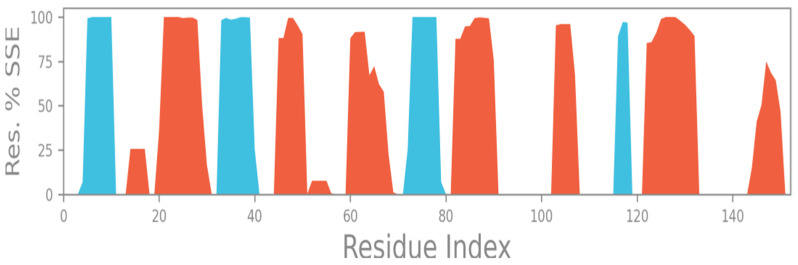
NDK Protein SSE measurement.

The SSE measure indicated the positional changes that occurred in the secondary structural elements of the protein, which were subjected to MD simulation. The SSEs of the NDK protein during the 100 ns simulation period were tracked, which revealed 32.41% Helixes and 14.75 strands, making up a 47.16% Total SSE, as indicated (helixes in red and strands in blue) (Figure 4).

### 2.6. Ligand RMSF

**Figure 5:**
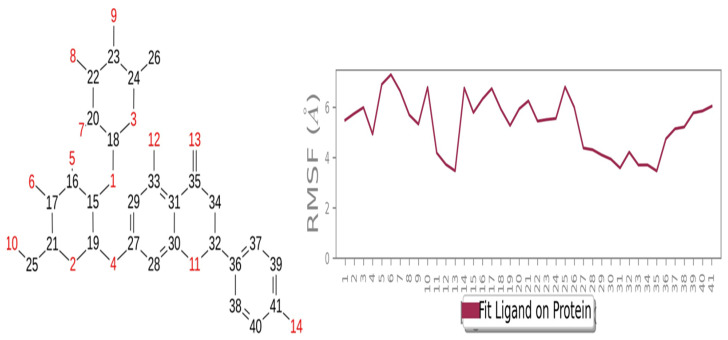
RMSF of Naringin Ligand.

The RMSF of Naringin indicated that it was made of 40 atoms, with some interaction atomic fluctuations in the atoms between 2 and 14. Ligand RMSF was found to be stabilized between 4 and 6 Å, which suggested a few atomic fluctuations (Figure 5).

### 2.7. Ligand Interactions

**Figure 6:**
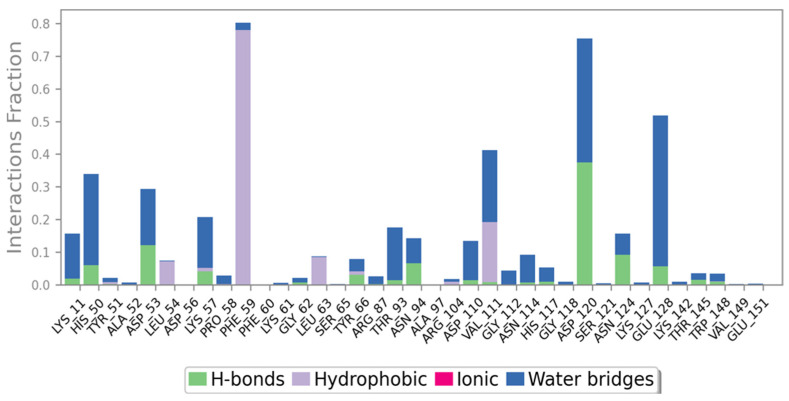
Ligand-Protein contacts with interaction fractions.

**Figure 7:**
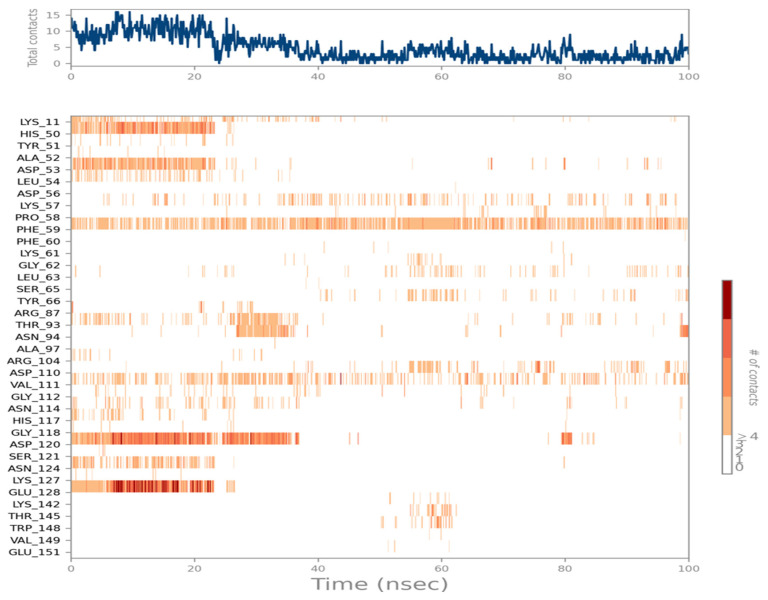
Ligand-Protein (Amino acid) contacts over a simulation duration of 100 ns.

The NDK amino acid residue ASP120 formed Hydrogen bonds, while PHE59 and VAL111 formed hydrophobic bonds with the Naringin molecule for more than 30% of the simulation time in the chosen trajectory (0.00 to 100.00 ns) (Figure 6). A timeline depiction of the communications and contacts can be observed in the graph with the upper panel depicting the total quantity of precise contacts between the protein and ligand and the second panel representing the residues that network with the ligand in each trajectory frame (Figure 7).

**Figure 8:**
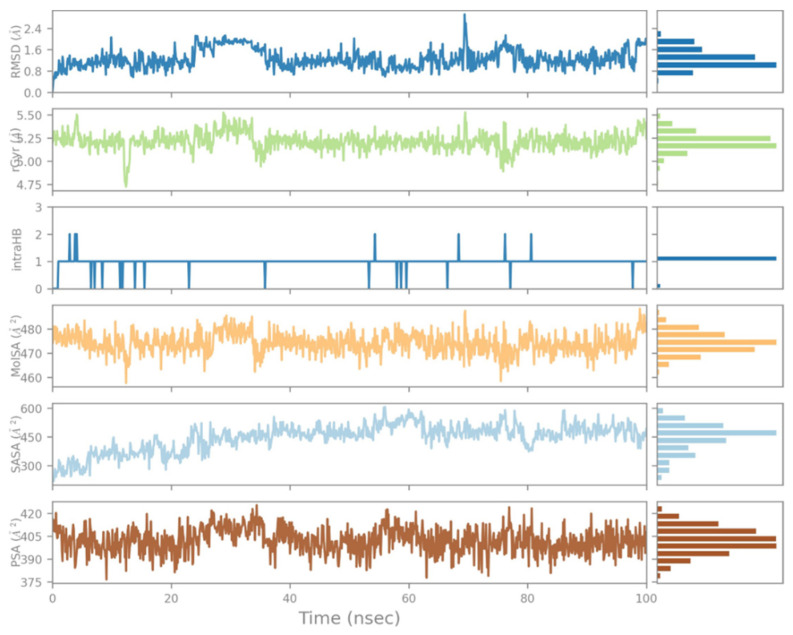
Ligand properties of Naringin.

The Solvent Accessible Surface Area during this interaction ranged between 300 and 600 Å^2^, while the polar surface area was between 375 and 420 Å^2^, and the radius of gyration (rGyr) ranged between 5.25 and 5.5 Å. The ligand RMSD varied from 0.8 to 2.4 Å during the simulation period of 100 ns (Figure 8).

### 2.8. ADME and Gastrointestinal Absorbability

**Figure 9:**
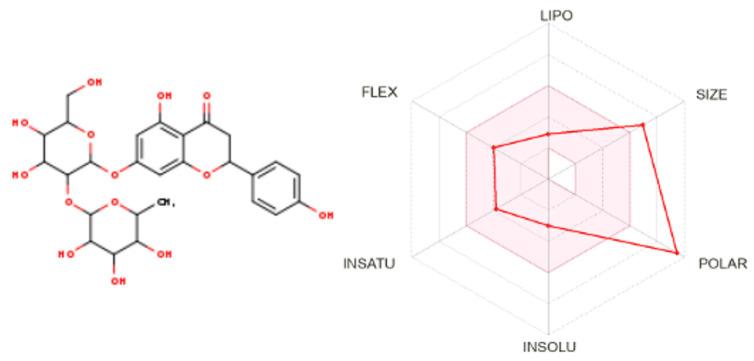
Structure and ADME characteristics of Naringin. The colored zone is the suitable physicochemical space for the oral bioavailability of Naringin.

The ADME and GI absorbability of the ligands throws light on the drug-likeness and safety of these molecules keeping in mind human and animal consumption. Of the selected phytochemicals in this study, epigallocatechin gallate, naringin, epicatechin gallate and rosmarinic acid were found to have a No/low gastrointestinal absorption. Meanwhile, o-coumaric acid and p-coumaric acid were predicted to have blood-brain permeability. Naringin (Figure 9) was observed to be orally bioavailable with very low intestinal absorption. It was found to be impermeable in the brain and skin. Naringin was a water-soluble molecule with a bioavailability score of 0.17 (Supplementary Table S1).

### 2.9. Boiled Egg Analysis

**Figure 10:**
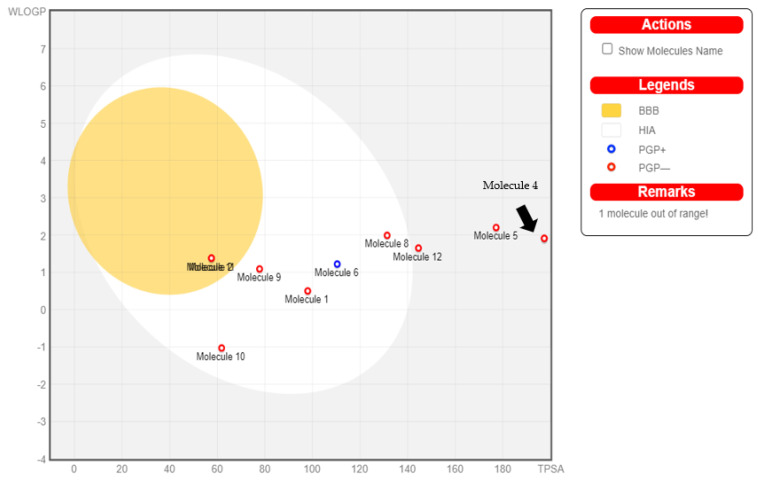
Boiled egg plot of the 12 selected coffee phytochemicals. Molecule 1—Gallic acid; Molecule 2—O-Coumaric acid; Molecule 3—Epigallocatechin gallate; Molecule 4—Naringin; Molecule 5—Epicatechin Gallate; Molecule 6—Catechin; Molecule 7—Gallocatechin gallate; Molecule 8—Quercetin; Molecule 9—Caffeic acid; Molecule 10—Caffeine; Molecule 11—p-Coumaric acid; Molecule 12—Rosmarinic acid. Points located in the yellow region indicate the blood–brain barrier permeability, whereas points in the white region indicate absorption in the GI tract. Naringin is out of both yellow and white regions, indicating no blood–brain barrier permeability and no GI absorption. Epigallocatechin gallate and Gallocatechin gallate were found to be out of range.

To reconfirm the ADME findings, we carried out the boiled egg analysis of the test molecules to understand their permeability through the blood–brain barrier and the GI tract. We found that the boiled egg analysis results were in concurrence with the ADME findings (Figure 10). Naringin was found to be out of range for both the yellow and white regions of the plot, indicating that the molecule was unabsorbable in the GI tract and could not penetrate the brain. Epigallocatechin gallate, epicatechin gallate and rosmarinic acid showed poor gastrointestinal absorption and brain access. Gallic acid, catechin, quercetin, caffeic acid and caffeine (found in the white region of the Boil-egg plot) were predicted to have a high probability of passive absorption in the gastrointestinal tract. O-coumaric acid and p-coumaric acid were found to be highly penetrable into the brain (found in the yellow region of the boiled egg plot).

### 2.10. Toxicity Prediction

**Figure 11:**
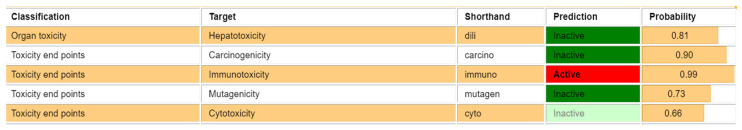
Toxicity prediction of Naringin.

To test the food-grade safety of Naringin to humans and animals, we carried out the toxicity prediction. It was found to belong to Class V of toxicity, meaning that it could be harmful only when swallowed with a median lethal dose between 2000 and 5000 mg/kg (Figure 11).

Furthermore, Naringin was also found to be non-hepatotoxic, non-carcinogenic, non-mutagenic and non-cytotoxic.

## 3. Discussion

Coffee has been a widely consumed plant variety across the globe. There have also been growing concerns regarding coffee waste management to achieve the sustainability of the coffee production chain [34,35]. Coffee pulp is the main by-product, which represents half of a coffee cherry [36]. It accounts for up to 29% of the total dry weight [36].

On the other hand, there are equally deep concerns regarding the management of foodborne fungi [37,38] and increasing events of antifungal resistance in them [39,40]. In particular, *A. fumigatus* has been repeatedly reported as resistant to first-line azole antifungal drugs. However, there is a limited understanding of the genetic mechanisms underlying the development of fungal resistance, which raises concerns about the potential emergence of opportunistic fungal superinfections [40]. The focus has now been on the identification of novel drug targets across unconventional pathways occurring across fungal biosystems to reduce the possibilities of infection while also keeping in mind antifungal resistance. The protein nucleoside diphosphate kinase (NDK) has been identified to be one such potential target owing to its importance in the nucleotide biosynthesis of the organism [20]. In a former study conducted by Sangta J, et al. (2021), phytochemicals recovered from the *Coffea arabica* pulp, which was identified using Quadrupole Time-of-Flight Mass Spectrometer reportedly demonstrated antifungal activity against *Alternaria brassicicola*, *Pestalotiopsis* sp. and *Paramyrothecium breviseta* [25,41]. A number of researchers have tried to ascertain the bioactive nature of *Coffea arabica* L., with an aim to identify the specific compounds that are responsible for the bioactivity. This plant has been observed to have nearly a 12% protein content, including the presence of polyphenols such as epigallocatechin gallate, naringin, epicatechin gallate, catechin, gallocatechin gallate, and quercetin, phenolic acids and caffeine alkaloids such as p-coumaric acid, caffeic acid, caffeine, rosmarinic acid, o-coumaric acid, and gallic acid with significant antifungal properties [25]. In continuation of these findings, we carried out the in silico screening of isolated phytochemicals for their antifungal activity against the *A. fumigatus* foodborne fungus, which is of great public health importance [41].

During this study, we found that the molecular docking interactions of selected phytochemicals against the NDK protein revealed successful interactions with the highest glide score of −6.771 kcal/mol for Naringin, followed by Rosmarinic acid (−6.072 kcal/mol). While Naringin and Epigallocatechin gallate both showed seven hydrogen bonding interactions, the glide score for the Epigallocatechin gallate–6XP7 interactions was −5.687 kcal/mol, and hence, Naringin was opted for further validation with molecular dynamic simulations. The lowest number of interactions was observed for Caffeine with 6XP7, demonstrating only two hydrogen bonds. The lowest glide score of −4.186 kcal/mol was observed for the caffeic acid–6XP7 complex. Hydrogen bonds, occurring between the ligand and proteins, have always been crucial in determining the specificity of observed interactions. Adding to this, interactions involving hydrogen bonds, pi–pi stacking, and salt bridges were found to be crucial when attributed to the increased inhibitory value of the ligand. The docking glide score and MD simulation, including RMSD and RMSF findings, indicated these interactions to be in a range from moderate to strong [42]. The molecular dynamic simulations of the Naringin-6XP7 complex revealed a stable protein–ligand complex throughout the entire duration of the simulation [29,43]. It also helped when studying the stability of the amino acid contacts in both the protein and Naringin through the simulated period. The study conducted by Santa et al. focused on the composition of the coffee pulp and employed high-performance liquid chromatography to analyze its components through which Naringin was identified as a constituent of the coffee pulp. The integration of this in silico approach with the experimental findings of Santa et al.’s research, could help validate and enhance the reliability of these findings, contributing to a more comprehensive understanding of the topic at hand. This experimental evidence strengthens the argument by providing empirical support for the presence of Naringin in coffee pulp and provides a basis for the further validation of the antifungal nature of this compound in vitro.

Naringin, a flavone glycoside, has been reported for its biomedical significance and for its antioxidant, anti-inflammatory, antiviral, bone regenerative, and potential to inhibit/reduce neurodegeneration, genetic damage, cardiovascular diseases, and human malignancies [44]. Naringin has demonstrated substantial antifungal action against *C. albicans* [45], *Botrytis cinerea*, *Trichoderma glaucum*, and *Aspergillus fumigatus* [46], *Aspergillus parasiticus*, *Aspergillus flavus*, *Fusarium semitectum* and *Penicillium expansum* [47]. Nonetheless, no recent studies have investigated the anti-*Aspergillus fumigatus* activity of the molecule to ascertain the underlying mechanistic basis to indicate a possible consideration toward its interactions with the NDK.

The ADME and gastrointestinal absorbability of Naringin indicated that the molecule was orally bioavailable with very low possibilities of intestinal absorption. This was further supported by the boiled egg analysis data, which clearly indicated that the GI absorption of the Naringin molecule was obscure. Drug-target molecules which target body parts other than the brain should ideally be impermeable to the brain to avoid psychotropic side effects. Naringin has been reported to be non-toxic for Sprague Dawley rats with no-observed-adverse-effect-level (NOAEL) in the molecule being >1250 mg/kg/day of the rodent upon exposure for 13 weeks [48] and 6 months [49]. In a similar study, naringin was demonstrated to have a good safety profile in beagle dogs with a NOAEL of at least 500 mg/kg/day when administered *per os* [50]. Naringin was also reported to be of low toxicity when employed as a sensory additive in animal feed [51]. In alignment with these findings, the toxicity prediction reports indicated that Naringin was safe for human consumption upon further investigation.

However, the findings of the current study should be validated with appropriate in vitro and in vivo antifungal experimentation, which could lead to the development of a plant-based non-toxic, antifungal agent to combat *A. fumigatus* infections.

## 4. Materials and Methods

The current study was performed using an in silico approach involving compound library preparation based on a review of the literature, molecular docking, molecular dynamics simulations, ADME (Absorption, Distribution, Metabolism, and Excretion), gastrointestinal (GI) absorbability, and toxicity evaluation for the lead molecule [35,52,53].

### 4.1. Molecular Docking

#### 4.1.1. Protein Preparation

**Figure 12:**
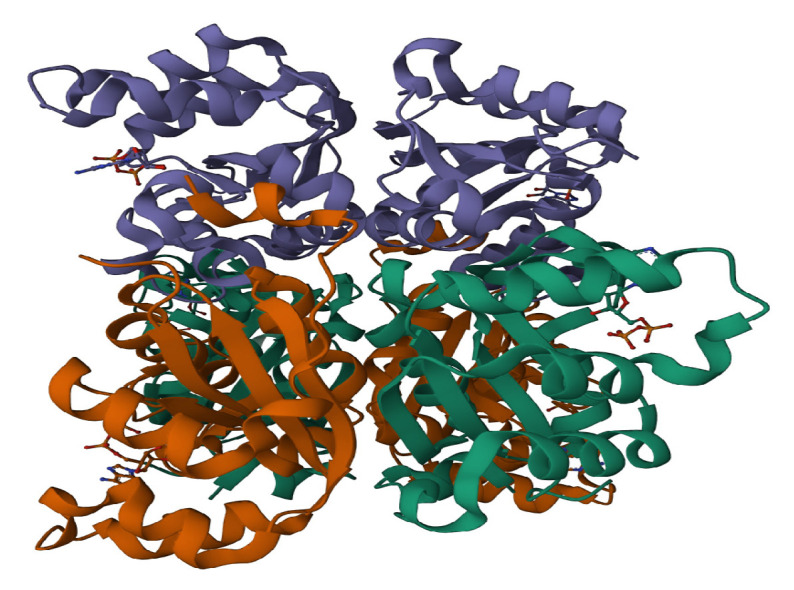
The 2-dimensional structure of *A. fumigatus* Nucleoside diphosphate kinase (NDK) protein. (PDB ID: 6XP7).

The crystal structure of the protein NDK from *Aspergillus fumgiatus* (PDB ID: 6XP7) (Figure 12) with a resolution of 2.2 Å, with 3 chains of a 162-length amino acid sequence was chosen from RCSB PDB and was obtained directly onto the Maestro workspace (V 13.1, Trial version issued by Schrodinger India to JSS Academy of Higher Education and Research, Mysore) from the Protein data bank. The protein preparation wizard panel, under GLIDE in Maestro V 13.1 of Schrodinger, was utilized to perform the assessment and refine the protein structure [54]. Chain A was retained and utilized for molecular docking studies. In addition to filling the missing loops and side chains using the PRIME module, the preparation of the protein structure included the removal of water molecules, the addition of hydrogen atoms, the assignment of bond order, and the deletion of other heteroatoms with the pH set at 7.5. Further, the optimization of the protein structure was carried out, and the energy minimization was achieved using an OPLS3e force field with a constraint of 0.30 Å RMSD.

#### 4.1.2. Receptor Grid Generation

The prepared protein chain was further used to generate a grid box. The grid box was defined around the pre-bound ligand in such a way that the center of the docked ligand was in identical dimensions to the binding box. Glide’s standard precision mode (SP) was employed for the rigid receptor docking technique, which was founded on the OPLS-3e force field and scaling factor 1.0 [54].

#### 4.1.3. Ligand Preparation

The *Caffea arabica* phytochemicals isolated and identified in previous research as per Sangta J, et al. (2021) were used to prepare a library of compounds for the in silico evaluation of anti-*A. fumigatus* properties. The structures of the compounds epigallocatechin gallate, naringin, epicatechin gallate, catechin, gallocatechin gallate, quercetin, caffeic acid, caffeine, p-coumaric acid, rosmarinic acid, o-coumaric acid, and gallic acid, were obtained in a .sdf format from Pubchem and were transformed into 3D structures using the Maestro Schrödinger 13.1 Ligprep module. The Ligprep assigned proper bond ordering and corrected the protonation and ionization states of the ligands. The minimization of energy was carried out at a stable pH of 7.5, with specific chirality.

#### 4.1.4. Molecular Docking

The protein was immobile, whereas the ligands were flexible during the docking process. A regular precision mode was used for docking. Molecular docking was executed after synthesizing the ligand and protein and defining the grid in place of the pre-existing ligand on the protein. GLIDE was used to dock the identified ligands with the protein’s X-ray crystal structure. The best compounds for each target were chosen based on their glide g score, thermodynamic ideal energy value, the types of interactions, bonding potential, and conformations.

### 4.2. Molecular Dynamics Simulation

Based on various parameters of molecular docking, the best-docked molecule was selected for further validation. This technique is a bioinformatic method that simulates the actual movements of the atoms in the protein–ligand complex for a set duration. It is used to study the movement of the protein–ligand complex in a solvent system. The best-docked model of the docked complex was used to achieve this using the DESMOND v 13.1 System Builder workflow. The best pose with an appropriate glide score obtained from molecular docking was further taken up for the molecular dynamics simulation using the DESMOND v 13.1 System Builder workflow. The boundary was set at 10 × 10 × 10 Å orthorhombic box, and the TIP3P water model of the solvent system was introduced using the OPLS4 force field. These proteins were neutralized with the addition of salt ions (sodium and Chlorine) in order to balance the overall charge of the system. A molecular simulation was run for 100 ns at the recording energy interval of a 12 ps simulation run. The whole system contained 20,692 atoms. The pressure and temperature were sustained at 1.01325 bar and 300 K, respectively. The results were analyzed with the help of a simulated interaction diagram tool that was built within the DESMOND panel to generate a report.

### 4.3. Prediction of ADME Properties

ADME (Absorption, distribution, metabolism and excretion) parameters, pharmacokinetic, drug-like properties, and the medicinal chemistry friendliness of selected phytochemicals were performed using the SWISS ADME tool by the Swiss Institute of Bioinformatics (http://www.swissadme.ch/index.php (accessed on 2 April 2023)). The canonical smiles of the 12 molecules were obtained from the Pubchem database and were added to the web browser and run for the calculation of ADME properties. Further, a boiled-egg plot of the 12 molecules was obtained from the same tool to identify and represent the molecules that had the capability to cross the blood–brain barrier and to be absorbed via the gut.

### 4.4. Toxicity Prediction

For the estimation of toxicity in small molecules, such as acute toxicity, hepatotoxicity, cytotoxicity, carcinogenicity, mutagenicity, and immunotoxicity, the ProTox-II tool by The Charite University of Medicine, Berlin, Germany (https://tox-new.charite.de/protox_II/ (accessed on 2 April 2023)) was used. These molecules were classified based on their toxicity ranging from Class I to VI and from fatal to non-toxic depending on the lethal dose calculations, which ranged from an LD50 value lower than 5 (Class I) to an LD50 value of more than 5000 (Class VI) The 6 classes of toxicity ranged from fatal if swallowed to non-toxic.

## 5. Conclusions

These findings were made as a part of this study, and earlier reports indicate that Naringin from coffee pulp is a good antifungal candidate that acts via the NDK pathway to inhibit *A. fumigatus*. Upon the necessary in vitro and in vivo investigations to ascertain the mechanistic basis of the observed bioactivity and toxicity, Naringin may be an ideal candidate to produce anti-*Aspergillus fumigatus* treatments and packaging material for foods and food products. Thereby, this could address the dual concerns of spent coffee waste management and antifungal resistance to the available first-line Azole drugs.

## Data Availability

Data are contained within the article or Supplementary Material.

## Supplementary Materials

The following supporting information can be downloaded at: https://www.mdpi.com/article/10.3390/molecules28135189/s1. Supplementary Table S1: ADME and Boiled-egg plot analysis of coffee phytochemicals.
