# Supplementary material for: Naringin from Coffee Inhibits Foodborne Aspergillus fumigatus via the NDK Pathway: Evidence from an In Silico Study
Source: Molecules. 2023 Jul 4;28(13):5189. doi: 10.3390/molecules28135189 (PMC10343685; doi:10.3390/molecules28135189)
Supplement: Supplementary file 1 [file molecules-28-05189-s001.zip › molecules-2441632-supplementary.pdf]

Supplementary Table S1: ADME and Boiled-egg plot analysis of coffee phytochemicals.

	Drug Likeness		Solubility	Absorption			Distribution					
Molecule	Lipinski	Bioavailability Score	Water Solubility	Intestinal Absorption	Skin Permeability (cm/s)	P-glycoprotein Substrate	BBB Permeability	CYP1SA2 Inhibitor	CYP12C19 Inhibitor	CYP2C9 Inhibitor	CYP2D6 Inhibitor	CYP3A4 Inhibitor
Molecule 1- Gallic acid	Yes	0.56	Yes	High	-6.84	No	No	No	No	No	No	Yes
Molecule 2- O-Coumaric acid	Yes	0.85	Yes	High	-5.86	No	Yes	No	No	No	No	No
Molecule 3- Epigallocatechin gallate	No	0.17	Yes	Low	-8.27	No	No	No	No	No	No	No
Molecule 4- Naringin	No	0.17	Yes	Low	-10.15	Yes	No	No	No	No	No	No
Molecule 5- Epicatechin Gallate	Yes	0.55	Yes	Low	-7.91	No	No	No	No	No	No	No
Molecule 6- Catechin	Yes	0.55	Yes	High	-7.82	Yes	No	No	No	No	No	No
Molecule 7- Gallocatechin gallate	No	0.17	Yes	Low	-8.27	No	No	No	No	No	No	No
Molecule 8- Quercetin	Yes	0.55	Yes	High	-7.05	No	No	Yes	No	No	Yes	Yes
Molecule 9- Caffeic acid	Yes	0.56	Yes	High	-6.58	No	No	No	No	No	No	No
Molecule 10- Caffeine	Yes	0.55	Yes	High	-7.53	No	No	No	No	No	No	No
Molecule 11- p-Coumaric acid	Yes	0.85	Yes	High	-6.26	No	Yes	No	No	No	No	No
Molecule 12 Rosamarinic acid	Yes	0.56	Yes	Low	-6.82	No	No	No	No	No	No	No
